# A historical review of calcaneal fractures: from the crucifixion of Jesus Christ and Don Juan injuries to the current plate osteosynthesis

**DOI:** 10.1007/s00264-022-05384-3

**Published:** 2022-03-25

**Authors:** Carlo Biz, Mariapaola Refolo, Felicia Deborah Zinnarello, Alberto Crimì, Federico Dante, Pietro Ruggieri

**Affiliations:** Orthopedics and Orthopedic Oncology, Department of Surgery, Oncology and Gastroenterology DiSCOG, University of Padova, via Giustiniani 2, 35128 Padova, Italy

**Keywords:** Calcaneus, Calcaneal fractures, Don Juan injuries, History, Osteosynthesis, Plating

## Abstract

**Purpose:**

Calcaneal fractures are one of the most challenging injuries to treat and one of the most divisive. The purpose of this historical review is to highlight the evidence of calcaneal fracture and its treatment through history.

**Methods:**

Archaeological, religious, artistic, literary and historical accounts were searched for descriptions of calcaneal fracture to give a thorough overview of the subject. The scientific literature was searched to highlight the evolution of treatment techniques.

**Results:**

For over 2500 years, the only available option was conservative treatment due to the high risk of infection and limb loss in a world without antibiotics, plastic surgery techniques and adequate osteosynthesis devices. At the beginning of the twentieth century, treatment was still rather crude, consisting of closed reduction by impaction by a Cotton’s mallet, immobilisation of the foot into presses and strict bed rest in a plaster cast for five weeks. Only in the case of untreatable pain, triple arthrodesis could be employed. Regardless, the results were dismal. The debate on the superiority of open reduction and primary subtalar arthrodesis over open and closed reduction spans the entire history of medicine.

**Conclusion:**

The long path of history has brought great improvement in the treatment of calcaneus fracture, but the debate about the best treatment is far from being over. There is a lack of good quality randomised control trials conducted according to an agreed set of outcome scores despite some excellent efforts. Therefore, despite the attempts made over the years and new, more precise prognostic scores, the outcomes of each technique in use today are as unique as the individuals who suffer from a calcaneal fracture.

## Introduction

Throughout history, calcaneus fractures have been considered nothing short of a curse. One of the oldest mentions of heel injury is found in the Bible. In the book of Genesis 3:15 (‘*Vulgata Clementina’*), God cursed the serpent in the following terms: ‘I will put enmity between you and the woman, and between your offspring and hers; he will crush your head, and you will strike his heel’ [1]. John, the beloved disciple, seems to summarise in one sentence of his Gospel the plan of God revealed through the Old Testament by Exodus 12:46, Numbers 9:12 and Psalm 34:20: ‘these things happened so that the scripture would be fulfilled: Not one of his bones will be broken’ (the Gospel of John 19:36). From some recent archaeological discoveries, it appears possible that Jesus Christ was crucified with a long nail through the calcaneus [2].

**Fig. 1:**
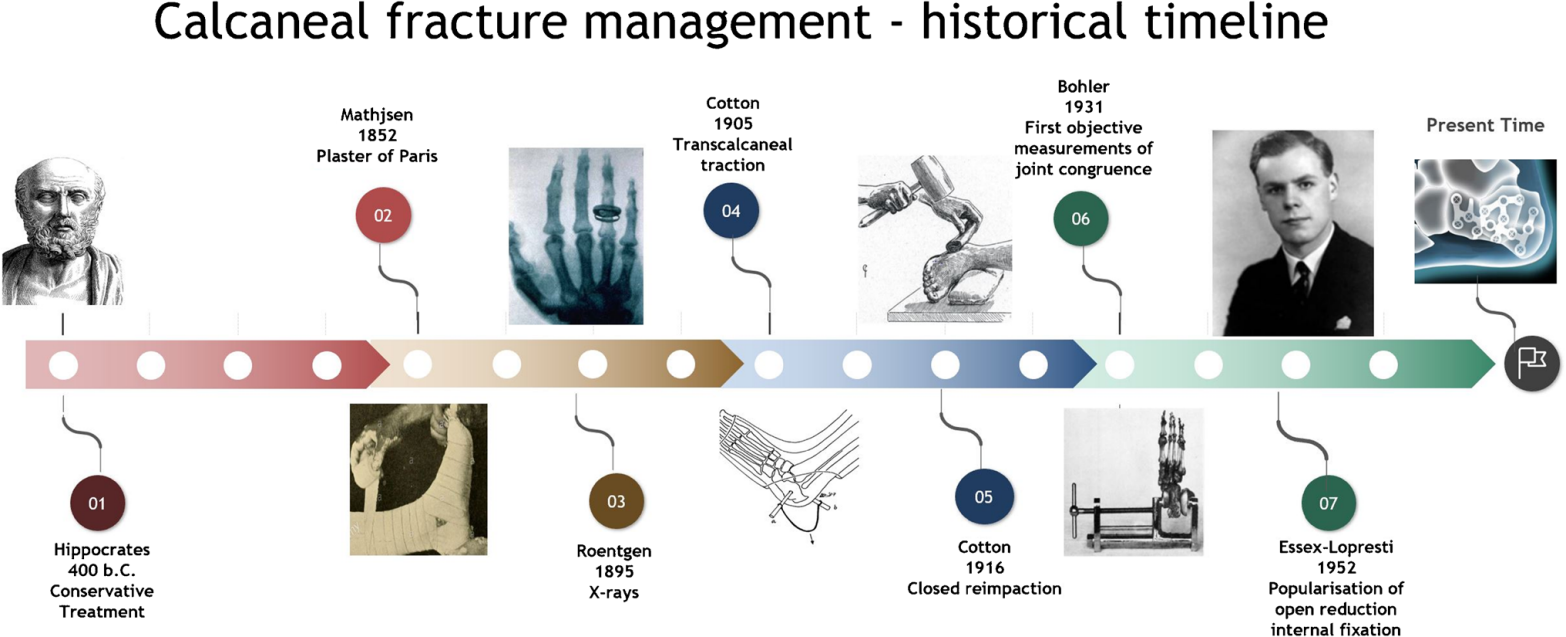
Calcaneal fracture treatment historical timeline

Whether divine or otherwise, the curse did find its way from the religious to the profane and the medical history of humankind. In the eighteenth century, the moniker ‘lover’s or Don Juan fractures ‘ took hold, referring to the classical mechanism of injury—a fall from height. Shortly after, the industrial revolution revealed the full destructive potential of this injury with the increase in new occupational hazards such as road traffic and aviation accidents. ‘Ordinarily speaking,’ F. Cotton in 1916 wrote, ‘the man who breaks his heel bone is ‘done’, so far as his industrial future is concerned’ [3]. The World Wars gave further impulse to the treatment of calcaneal fractures through countless ballistic and explosive-related injuries, leading eventually to the creation of more and more technologically advanced methods of osteosynthesis (Fig. 1) [4, 5].

## Definition and historical importance

**Fig. 2:**
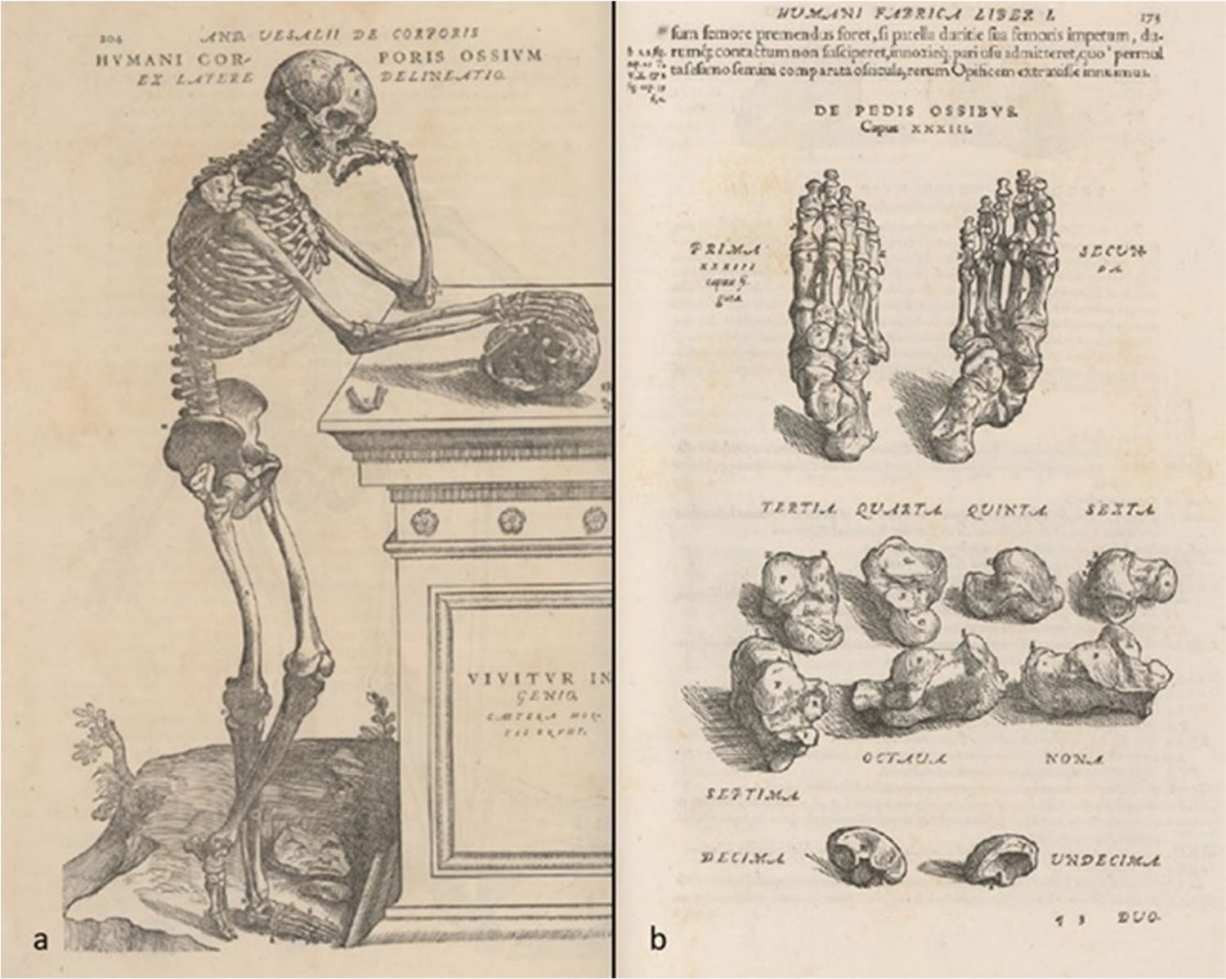
**a** Meticulously illustrated human skeleton; **b** Illustration of the skeleton of the foot; by Andreas Vesalius (1514–1564) in *De Humani Corporis Fabrica* *,* *libri septem* (p. 204 and p. 173)

The calcaneus or os calcis [6] is the structure through which most of the body weight is transmitted during locomotion. Both names, respectively attributed to Galen and Celsus [7], derive from *Calx-Calcis*—‘limestone’ in Latin—a loan from the Greek χάλιξ, which indicates a pebble or gravel [8]. Its anatomy was accurately described and illustrated by Andreas Vesalius (1514–1564) in the *De Humani Corporis Fabrica Libri Septem* (Fig. 2), published in 1543 [9].

Calcaneal fractures account for 2% of all foot and ankle trauma presentations, usually through an axial load caused by a fall from height or other high-energy trauma. The loading force impressed onto the talus which, acting as a wedge, transmits it through the calcaneus, constituted mostly of cancellous bone enveloped by a relatively thin cortex perforated by only a few branches of the posterior tibial artery, dorsalis pedis and perforating peroneal artery. These branches constitute variable extraosseous anastomoses vulnerable to ischemia and complications such as osteomyelitis, avascular necrosis and post-traumatic osteoarthritis [10, 11].

The oldest archaeological evidence of a calcaneal fracture comes from the remains of a hominin, excavated in Sterkfontein, who had sustained a crush fracture with comminution and wedging of the calcaneus into the talus. The finding suggests that this injury has been relevant to human survival. A broken heel meant the impossibility to escape predators and certain death [12].

The earliest written historical record on the natural history and treatment of this injury dates to Hippocrates in his vivid description of how heel fractures led to ‘very acute fevers […] tremblings, hiccup, aberration of intellect […] which prove fatal within a few days’. He described his treatment approach with ‘the foot […] raised a little higher than the rest of the body […] such patient will get well in sixty days if he keeps quiet’ [13].

There is no convincing record of premodern attempts at surgical management. A small clue came recently from a far related research field: the pathology of crucifixion. Traditionally, Jesus Christ is portrayed with a long nail driven from anterior to posterior with one foot atop the other.

However, this technique would require greater control by the executioner to keep one foot on top of the other while driving the nail [14]. It seems that the iconographic practice of representing the feet of Jesus crucified separately—that is, fixed by two different nails—was widespread until the twelfth century. Crucifix n. 432, the ‘Master of the Cross’ (1180–1200), part of the Gallerie degli Uffizi collection, is a perfect example of this modality, still formally connected to the Byzantine typology of the ‘Christus Triumphans’. Only much later artists started favouring the iconographic typology of the two feet nailed one over the other, as can be observed in a magnificent crucifix initially attributed to Donatello, dated towards the end of the fifteenth century.

Countless images of Jesus’s sacrifice have literally enshrined the concept, corroborated in the eyes of the believer by the holy relics brought as proof. Among these is a 12.5-cm-long and 9-mm-thick square nail, with a bell-shaped head tapering to 5 mm towards the end. It is held in the Basilica di Santa Croce in Jerusalem, in Rome. Saint Helen, Emperor Constantine’s mother, acquired it as the nail from the Holy Cross.

**Fig. 3:**
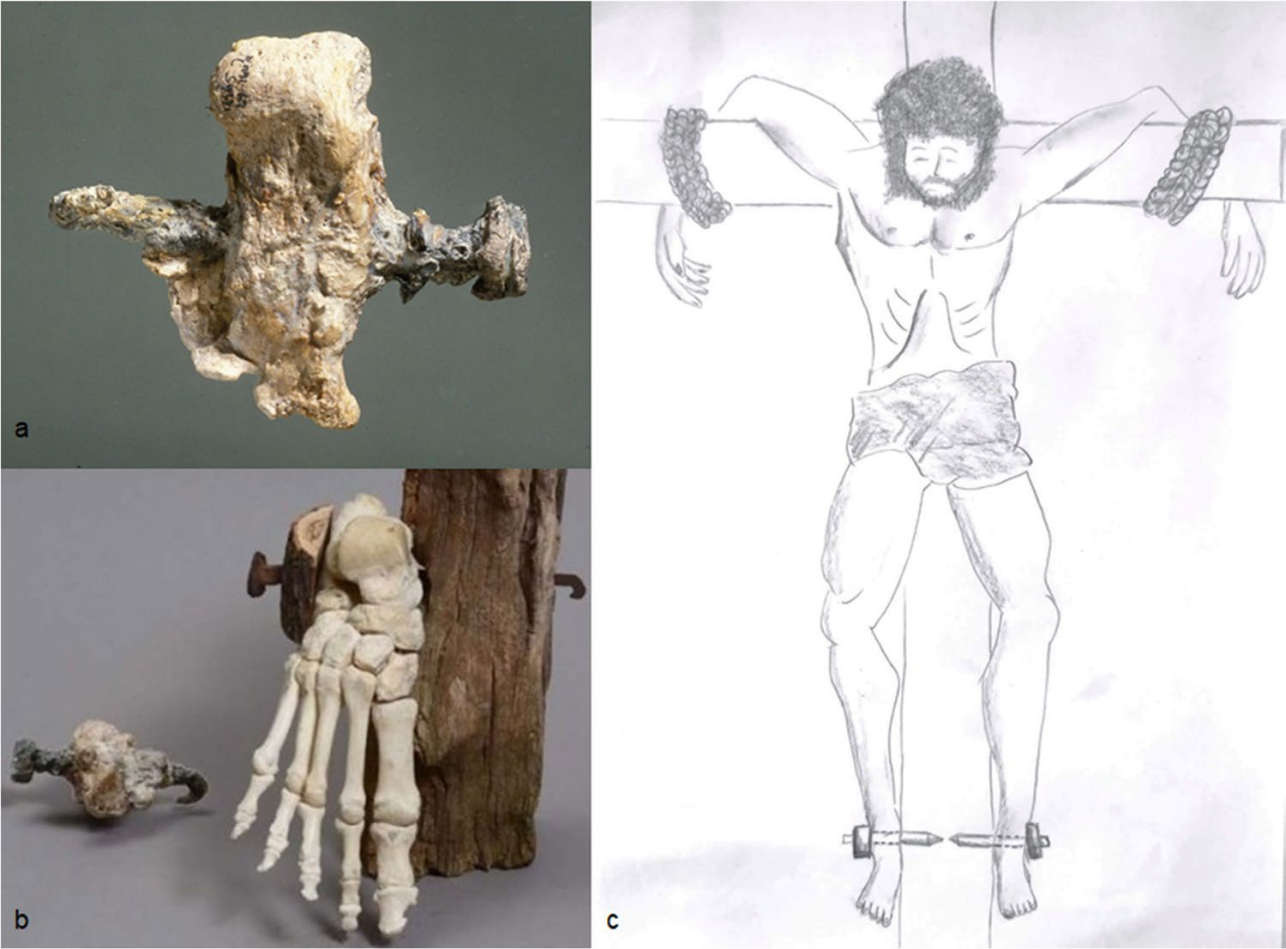
**a** Heel pierced by an iron nail in a man put to death by crucifixion dated first century CE. Found in 1968 in north Jerusalem in an ossuary under the name ‘Yehohanan son of Hagkol’, Israel Museum, Jerusalem. **b** Reproduction of the crucifixion using a nail through the calcaneus. **c** Illustration of how the victim is nailed to a large wooden beam

However, there is no convincing proof of crucifixion as a mechanism of calcaneal fracture by transfixion: so far, only one specimen was excavated in 1968 in Givat HaMivtar, Israel, a long nail transfixed in the calcaneus of a young man from lateral to medial [2]. This might recall the lateral surgical approach to the calcaneus, as if the executor wanted to avoid injury to the tibialis posterior artery. From one such specimen, it is impossible to draw any conclusions, either on the modalities of crucifixion or on the treatment of calcaneal fractures (Fig. 3).

**Fig. 4:**
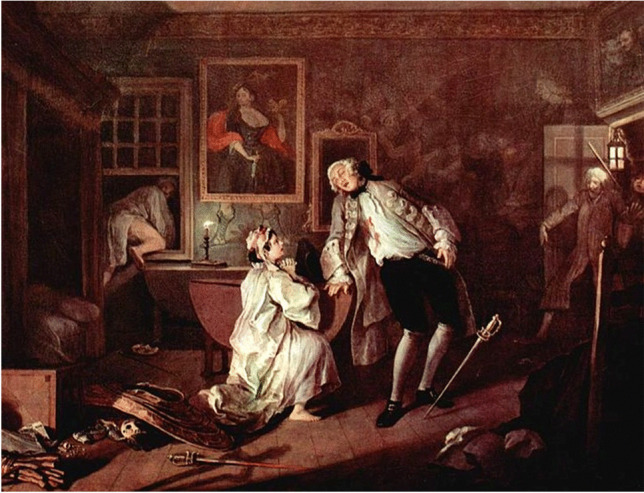
‘The Bagnio’, William Hogarth (London 1697–1764), Marriage A-la-Mode, scene n.5 (1743), oil on canvas, 70.5 × 90.8 cm, The National Gallery, London, UK; a lover makes an undignified hasty exit from a window, having been caught in flagrante delicto by his mistress’ husband

History remains silent on the issue until the eighteenth century, during which the moniker ‘the lover’s fracture ‘ or Don Juan fractures appeared to describe the most scandalous injury, likely inspired by DeSault. In his memoir, he tells of ‘a man, likely to be arrested by someone who pursued him’, who ‘leapt from a window nearly twelve feet high’, whose ‘feet struck on a beam which lay in his way’, causing a fall from which ‘he was unable to rise again’ [15]. The moniker is a jibe, popular in English-speaking countries, indicating heel fractures suffered by those presumed to have escaped the wrath of a husband by jumping from a window, as portrayed in the well-known painting ‘The Death of Him’, by William Hogarth (Fig. 4) and more recently depicted in a modern key by the British Bansky (1973 or 1974).

Heel fracture was formerly considered rare just ‘because it was not recognized’ [16]. The industrial revolution brought these injuries and their grim prognoses to the forefront due to their economic implications, as ‘ordinarily speaking […] the man who breaks his heel bone is ‘done’, so far as his industrial future is concerned’ [3]. Despite the life-changing consequences of such injuries, little changed in the way of treatment for many centuries.

### Splint immobilisation and reduction by traction

Over the past three centuries, the debate on calcaneal fractures management ebbed and flowed, from the watch and wait, no-touch position towards more invasive approaches, only to swing back again to closed methods in the face of poor results.

The French surgeons of the eighteenth century paved the way for later endeavours. Garangerot, in 1720, recommended bed rest to manage the ‘smash fracture […] until fragments had consolidated’. Petit, who trained under DeSault in the first half of the eighteenth century was more optimistic. In his view, all types of calcaneal fractures, ‘if properly treated […] will terminate as favourably as those of other bones’ [17]. The mainstay of treatment was a closed reduction by foot extension and application of the DeSault apparatus, which consisted of a ‘thick compress, not very broad, laid transversely above the fragment, secured by the long roller, and afterwards by a circular bandage […] a kind of figure of eight around the fracture’, holding the limb in elevation [15].

In the 1820s and 1830s, Cooper in England and Bérard and Bouchet in France used different splinting techniques, aware of the dangers of employing such treatment in the presence of neurovascular damage [18–20]. As of 1880, Bailey still recommended ‘elevation, lotions, saline cathartics and no splints’, an advice that rings appropriate to this day, as it recognised the small but real risk of compartment syndrome [21].

**Fig. 5:**
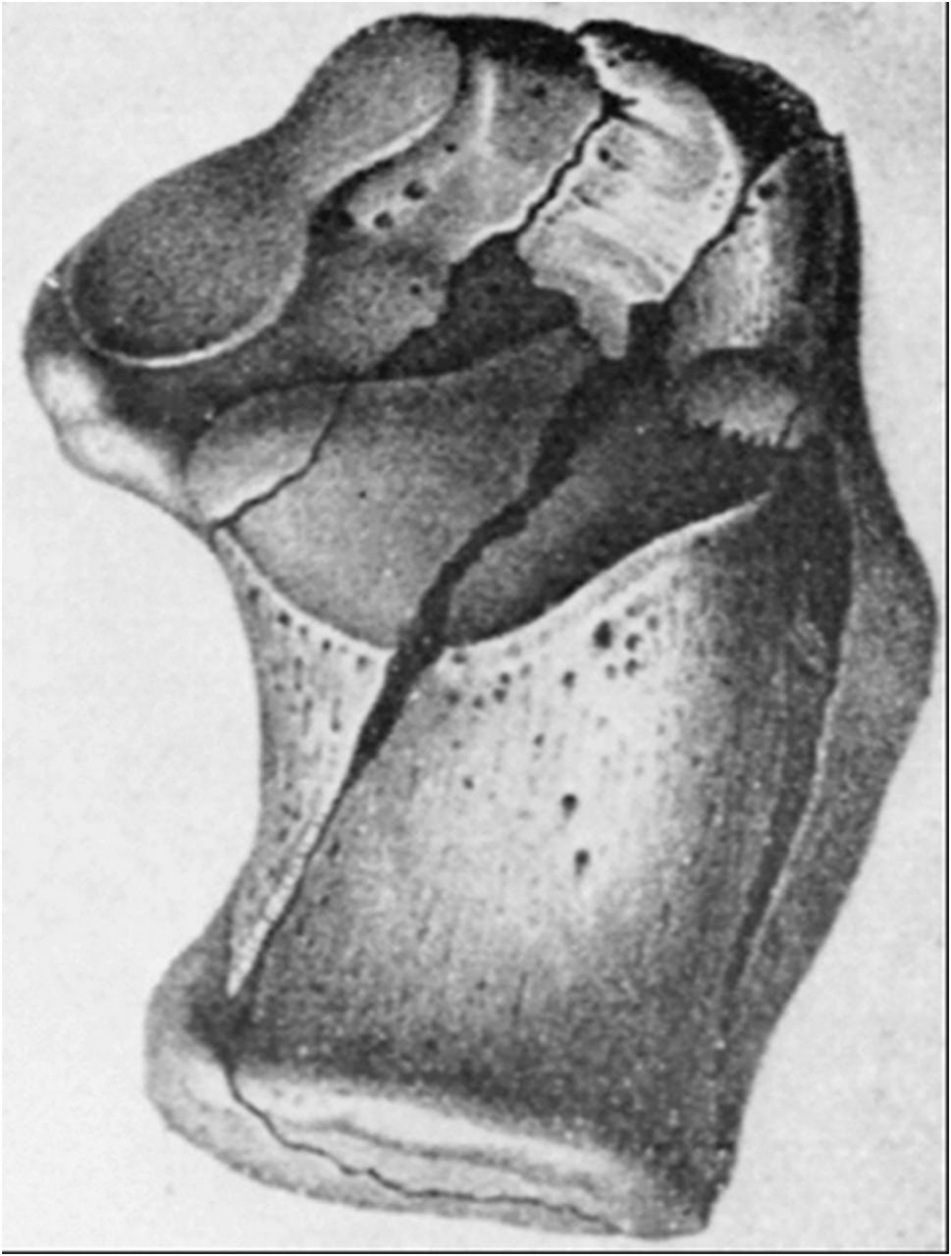
Intra-articular calcaneal fracture with the typical fragmentation seen from the superior aspect, Malgaigne JF. (1856) Die Knochenbrüche und Verrenkungen. Stuttgart: Reiger

The earliest systematic description and classification was published by Malgaigne in 1843, followed by his Atlas Landmark in 1856 in which he described the anatomy of calcaneus fractures with such precision as to compete with the most recent studies based on CT scan, and making the earliest attempt at classification (Fig. 5) [22].

However, these fractures were not completely diagnosed until the development of plain radiographs in the late 1890s [21].

There were no advancements in management approach though until the publication of the Charles Bell method in 1882, which consisted in ‘reduction in a lint-packaged splint and the instillation of the wound with phenol’, followed by ‘rest until consolidation took place’. [22].

The first attempt at reduction by traction is credited to L.G. Clark, an American surgeon. In 1855, he reported on immobilisation with a gutta-percha splint and weights applied to the posterior fragment by means of pulleys [23].

The advent of roentgenography in 1895 and plaster of Paris casting was nothing short of a revolution, allowing for the monitoring and control over anatomical reduction. Eisendrath in 1905 attempted a series of experimental open reductions and fixations augmented with kangaroo tendon grafts, followed by immobilisation in non-weight-bearing plaster. In 1908, Cotton used controlled traction and distraction of the fracture fragments by means of a device of his own creation, an apparatus consisting of a steel pin encircling the calcaneum passing behind the Achilles tendon, connected to weights by means of a Thomas Splint [24].

The poor results from available treatment and the accompanying severe consequences led surgeons such as Van Stokum in 1911 and Cahill in 1917 to pioneer approaches such as thalamic decalage with arthrodesis of the subtalar joint [23, 24].

Cahill is credited with a particularly bold attempt at osteosynthesis, with a screw in the great tuberosity of the calcaneus placed to achieve compression [25].

Despite the efforts, the shared opinion was that operative fixation was difficult and possibly even futile. The recommended course of action was closed reduction by impaction as described by Cotton in 1916 [3].

By the first half of the twentieth century, trans-calcaneal traction according to Clark, with some variations, was the mainstay of treatment [23]. In 1920 and 1922, Foldes and da Kaess, followed shortly after by Carabba and Gillette, expanded their apparatus with mixed outcomes [26, 27]. Their tractions and splints were cumbersome, forcing the patient to prolonged bed rest with all of its complications.

### The dawn of ORIF (open reduction and internal fixation) and Bohler’s angle

The real breakthrough came between 1921 and 1954 with the work of Leriche and Judet who pioneered open reduction and internal fixation of displaced intra-articular calcaneal fractures with staples and screws and even defect filling with autologous bone grafts [28, 29].

In 1927, Wilson documented open reduction and subtalar arthrodesis through lateral approach on 26 patients with excellent results in fresh fractures [30]. In 1928, Lenormant explained that defects created during open reduction and internal fixation of intra-articular fracture of the calcaneus can be filled with bone grafts [31].

Building on Leriche’s experience, Simon and Stulz in 1929 experimented with the application of generic plates after peroneal tendon division, followed by protected weight-bearing in a plaster cast [32].

In 1931, Bohler produced his study based on his extensive experience in treating foot trauma during World War I. Through his work, he showed that simple anatomical reconstruction is not a guarantee of functional recovery. Instead, the prerequisite for a good outcome is the reconstruction of the articular surface congruence. He thus described an objective X-ray measurement of articular congruence, the ‘tuber-joint angle’ that bears his name, still in use today for the evaluation of calcaneal fracture displacement and post-surgical reduction [33]. The importance of his work rests in the conclusion that anatomical reduction is indeed the key to a good outcome [34].

Regarding his contributions towards new techniques, he designed a vice for the compression of the lateral and medial wall. The results in terms of anatomical reduction were good if the vice was in place. Upon its removal, reduction was invariably lost. From this starting point, he elaborated further on distraction techniques by means of continuous trans-calcaneal traction using a Braun frame in an attempt to regain calcaneal length while achieving reduction. It did not solve the issue of prolonged non-weight-bearing, however, as his regimen included a four to five week immobilisation period with the traction pin in situ in a plaster cast, adding a further risk of deep infection [35]. His greatest contribution eventually came with the publication of his classification description based on X-rays, which constituted the departure point for all modern classifications, Sander’s included [34].

Around the same time, other illustrious surgeons were experimenting and refining closed reduction techniques. The Gissane technique, originally devised in Germany by Westhues, consisted of a variation of percutaneous pinning of the calcaneal tuberosity to achieve reduction followed yet again by plaster immobilisation. He created a tool that is custom fit for the reduction of calcaneal fractures known as the Nail of Gissane. This tool is still available to the foot and ankle surgeon as part of the modern posterior fragment osteosynthesis set [36].

By 1938, Goff had no less than forty-one different operative treatment methods to describe in his excellent review [16]. Despite all efforts, however, results of the treatment of these ‘serious and disabling injuries’ were still ‘incredibly bad’ due to the high infection rates and the inadequacy of tools and devices. These are the terms used by Conn in his work published in 1935 in which he accurately described the post-traumatic flatfoot deformity consisting of ‘pronated heels, planus arches and valgus forefoot with pain’ [35].

His approach was to take the healed, malunited fracture and restore position and alignment by performing a triple arthrodesis, with excellent results. Other surgeons were declaring defeat by advocating for delayed primary triple or secondary subtalar arthrodesis. This attitude almost brought calcaneus surgery to a halt in the mid-twentieth century.

A decade later, in 1943, Gallie was still recommending subtalar arthrodesis for healed fractures. Triple arthrodesis was reserved for those patients who had ongoing pain due to severe comminution of the talar-calcaneal joint or suboptimal reduction by means of the closed technique [37].

The Second World War revived the interest in calcaneal fracture treatment. In 1948, Kocher proposed an open reduction technique via lateral approach with autologous bone graft augmentation from the Iliac crest. In the same year, Palmer tested the approach. He showed that all patients were able to return to their previous occupations, with 90% reporting excellent results [38].

In 1953, Essex-Lopresti published a seminal article appraising the Westhues-Gissane method of percutaneous reduction and Palmer’s approach. Regarding the latter, 91% of patients had benefited from Palmer’s technique, returning to paid work in less than one year, but these results unfortunately could not be replicated. Based on his experience, Essex-Lopresti was able to recommend the use of Palmer’s approach for joint depression fractures only. He preferred his own treatment for tongue-type fractures consisting of percutaneous reduction with a Steinmann pin passed through the proximal calcaneal tuberosity, reduction of the fracture and synthesis with cannulated screws reaching into the calcaneocuboid joint with the foot in a plantigrade position [36].

In 1958, however, the controversy reignited and open techniques were under scrutiny again. According to McLaughlin, attempts at calcaneal fracture fixation were as futile as ‘nailing a custard pie to the wall’ [39].

Lindsay and Dewar in 1958 corroborated his view by showing that the non-operative approach was superior to operative treatment [40]. It proved that, despite its obvious advantages, the incidence of complications after open techniques was still too high.

This view dominated the landscape for another 20 years until the actual turning point with the advent of antibiotics, the production of appropriate hardware, the advancements in orthoplastic reconstruction and the superior diagnostic specificity of computer tomography, ultimately allowing for accurate anatomical reconstruction. Numerous studies on severity scores and classifications arose, offering clear indications for operative planning [39].

**Fig. 6:**
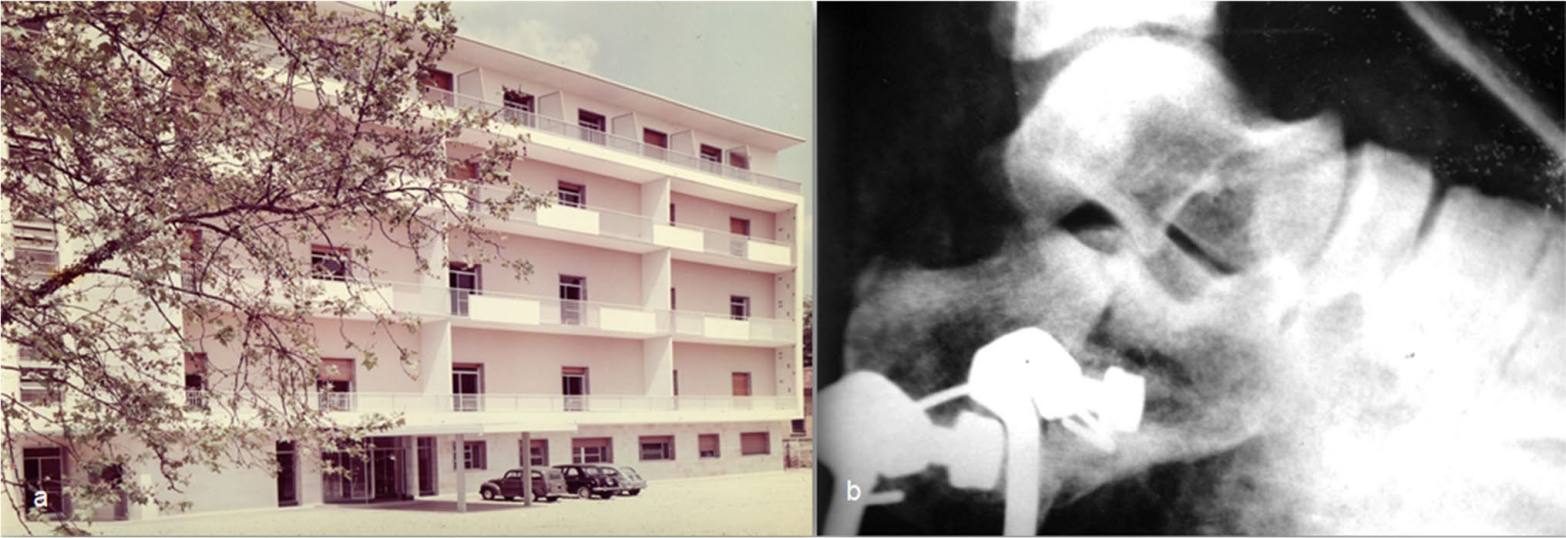
**a** Historical photo of the Orthopaedic Department of Padua 1959. **b** Intraoperative X-ray of a displaced multi-fragmented fracture with mixed sinking: one circumscribed fragment sunken vertically, the other horizontally treated with reduction by a trans-calcaneal wire (Orthopaedic Department of Padua, 1959)

Until the first half of the 1960s, Italian surgeons had employed a variation of the Bohler technique as perfected by Scaglietti [35]. It consisted of a closed reduction with vices and impactors, trans-calcaneal traction with a Kirschner wire and a rubber band tension wire applied onto a stirrup encased in a long plaster cast with 90° knee flexion and the foot in equinus position (Fig. 6).

The force intensity and direction could then be modified according to clinical and radiological follow-up to avoid fracture fragment depression. After 4 weeks of treatment, the traction was removed, and the cast, including the heel with the trans-calcaneal Kirschner wire in situ, was removed after 8 weeks.

## Contemporary osteosynthesis: general principles

**Fig. 7:**
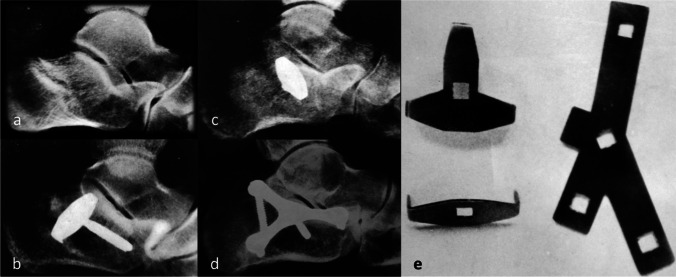
Radiographs of multi-fragmentary thalamic fractures treated with different synthesis systems: **a**) example of a thalamic fracture with sinking fragment; **b**–**c**) reduction and synthesis with a straight plate with two anchor points and a screw **d**) reduction and fixation with a Y-screw plate and screws; **e**) straight plates with two and three anchor points respectively (to the left), and a Y-screw plate (to the right) used at Orthopaedic Department of Padua, 1965

In the 1970s, French and Italian surgeons undertook large volume studies on open reduction and lateral plate osteosynthesis (Fig. 7). The mainstay treatment technique for depressed talar fractures was based on the Judet method, consisting in exposing both the fracture and the joint line to achieve perfected reduction and apposition of the articular surfaces. The construct was then held in place with a pronged plate and a screw supporting the thalamus, and bony defects were filled with autograft [41].

In the same years, new instrument and plate designs contributed towards a rigorously anatomical reduction.

**Fig. 8:**
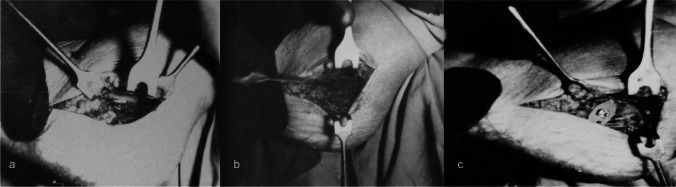
Intra-operative images of a multi-fragmentary fracture treated with plate and screws: **a** arthrotomy showing the sunken thalamus and the inferior talar articular surface; **b** lifting and reduction of the thalamus, and removal of small fragments of the external cortex; **c** synthesis of the fracture with a metal plate with two points to be inserted in the intact cortex and put in compression with a screw (Orthopaedic Department of Padua, 1965)

According to the Padua School, the preferable method was based on the Judet techniques, such as fixation with splintage with a bone graft and screws, a small straight plate and a screw, or a three-armed plate with screws (Fig. 8).

Calcaneal fracture treatment was still a controversial topic as, despite accurate reduction and reconstruction of the Bohler angle, the subtalar joint could still lose reduction. Eventually, the Bohler and Scaglietti methods were found to produce anatomically and clinically unsatisfactory results with a high incidence of complications such as loss of reduction and K wire insertion site infection. Open fixation had the advantage of offering greater anatomical control, albeit not always resulting in good clinical outcomes.

In 1975, Robert, Seour and Remy described an open reduction of thalamic fractures of the calcaneus by rotation and use of Kirschner wires for fixation [42]. Non-depressed thalamic fractures were routinely managed with immobilisation in a Griffin-type plaster cast for five to eight weeks followed by intensive physiotherapy or using percutaneous screws. Depressed thalamic fractures were deemed not amenable to closed treatment, as the rate of re-operation for unsatisfactory results was high [43]. Between 1970 and the mid-1990s, the advent of computer tomography with its superior diagnostic specificity and possibility of accurate anatomical reconstruction resulted in a significant leap forward, generating data for specific severity scores and classifications, offering clear indications for operative planning.

In recent times, the Padua school has been concentrating on the outcomes of percutaneous fixation with two or three titanium cannulated screws. The technique consists of closed reduction and provisional stabilisation of the fragments with 2-mm K-wires driven from the calcaneal tuberosity towards the subtalar joint used as joysticks to manipulate the fragments until restoration of Bohler’s angle is achieved under an image intensifier. Final stabilisation with titanium cannulated screws inserted in posterior-anterior direction is occasionally complemented with a latero-medial screw to better support the thalamic region, trying to minimise the chances of screw protrusion. Patients are kept non-weight-bearing for four weeks with passive and active ankle ROM exercises after 15 days [4].

With the introduction of specifically designed plates, such as the AO/ASIF plate, the osteosynthesis of multi-fragmented intra-articular fractures via the extensile lateral approach became the gold standard in the treatment of complex fractures. The conservative techniques championed by Westhues and Essex-Lopresti laid the foundation of contemporary percutaneous reduction in less complex fracture configurations. Further to this, sensible management of soft tissues and the judicious use of antibiotics under specialist guidance have been a major contribution to outcome. Despite the high incidence of post-traumatic deformity and osteoarthritis, the outlook is less grim, at least for younger men and women with a light workload. The foot is immobilised and elevated before surgery up to seven days, allowing for oedema resolution and soft-tissue preservation. Physiotherapy can start immediately after surgery with passive and active mobilisation, toe touch weight-bearing protected by crutches. Most patients are able to return to active employment in four to six months [44].

## Conclusions

Currently, the debate is far from being over about the best treatment for calcaneus fracture. There is a lack of good quality randomised control trials conducted according to an agreed set of outcome scores despite some excellent efforts [45]. A recent meta-analysis could only conclude that better anatomical results need to be balanced with the higher incidence of complications of open techniques [46]. In April 2021, a newer meta-analysis comparing ORIF to primary subtalar arthrodesis suggests that osteosynthesis for Sander’s type II and III fractures seems to give better outcomes two years post-operatively with respect to primary subtalar arthrodesis [5]. Therefore, despite the attempts made over the years and new, more precise prognostic scores, the outcomes of each technique in use today are as unique as the individuals who suffer from a calcaneal fracture. In the absence of conclusive evidence, treatment needs to be personalised through careful patient selection and exhaustive informed consent.

## Data Availability

Any research materials of this study are available at our institution and can be accessed.
